# Red Blood Cell Transfusion Need for Elective Primary Posterior Lumbar Fusion in A High-Volume Center for Spine Surgery

**DOI:** 10.3390/jcm7020019

**Published:** 2018-01-30

**Authors:** Giuseppe Ristagno, Simonetta Beluffi, Dario Tanzi, Federica Belloli, Paola Carmagnini, Massimo Croci, Giuseppe D’Aviri, Guido Menasce, Juan C. Pastore, Armando Pellanda, Alberto Pollini, Giorgio Savoia

**Affiliations:** 1 Neurosurgery I, Neuro Center, Humanitas Research Hospital, Via Manzoni 56, 20089 Rozzano, Italy; sm.beluffi@libero.it (S.B.); matiemia@gmail.com (F.B.); pcarmagnini@alice.it (P.C.); qq6tu@outlook.it (M.C.); giuseppe.daviri@gmail.com (G.D.); guidomenasce@tiscali.it (G.M.); juancarlospastore@gmail.com (J.C.P.); arpella@alice.it (Ar.P.); albertopollini@tin.it (Al.P.); giorgiosavoia@tiscali.it (G.S.); 2 Management Control Unit, Humanitas Research Hospital, Via Manzoni 56, 20089 Rozzano, Italy; dario.tanzi@humanitas.it

**Keywords:** blood, blood loss, spine surgery, spine fusion, lumbar, transfusion, red blood cell, hemoglobin, predictors, operative time

## Abstract

(1) Background: This study evaluated the perioperative red blood cell (RBC) transfusion need and determined predictors for transfusion in patients undergoing elective primary lumbar posterior spine fusion in a high-volume center for spine surgery. (2) Methods: Data from all patients undergoing spine surgery between 1 January 2014 and 31 December 2016 were reviewed. Patients’ demographics and comorbidities, perioperative laboratory results, and operative time were analyzed in relation to RBC transfusion. Multivariate logistic regression analysis was performed to identify the predictors of transfusion. (3) Results: A total of 874 elective surgeries for primary spine fusion were performed over the three years. Only 54 cases (6%) required RBC transfusion. Compared to the non-transfused patients, transfused patients were mainly female (*p* = 0.0008), significantly older, with a higher ASA grade (*p* = 0.0002), and with lower pre-surgery hemoglobin (HB) level and hematocrit (*p* < 0.0001). In the multivariate logistic regression, a lower pre-surgery HB (OR (95% CI) 2.84 (2.11–3.82)), a higher ASA class (1.77 (1.03–3.05)) and a longer operative time (1.02 (1.01–1.02)) were independently associated with RBC transfusion. (4) Conclusions: In the instance of elective surgery for primary posterior lumbar fusion in a high-volume center for spine surgery, the need for RBC transfusion is low. Factors anticipating transfusion should be taken into consideration in the patient’s pre-surgery preparation.

## 1. Introduction

Lumbar spine fusion is a common surgical procedure used for the treatment of many spinal conditions including those of a degenerative nature. Demand for spinal fusion presented a significant volume increase in operative neurosurgery over the last decades, with a 220% rise in the 1990s and a further increase of 12,000 procedures per year in the United States in the 2000s [1,2,3].

Unfortunately, significant blood loss remains a major concern in spine surgery, with a reported volume loss ranging from 0.65 to 2.1 liters per case [4,5,6,7,8,9]. Thus, lumbar spine fusion has been recognized to be among the top 10 surgical procedures that necessitate blood transfusion, with a rate that increased from 4% to 8% in the United States from 2000 to 2009, and further up to 18% in many instances during the most recent years [10,11,12]. Excessive blood loss may require allogenic blood transfusion in order to avoid morbidities, i.e., hypotension and hypoperfusion with cardiac, pulmonary, and renal damage, as well as coagulopathy [9]. Nevertheless, blood transfusion has been reported to be associated with higher risk of the development of wound infections, venous thromboembolism, pulmonary embolism, myocardial infarction, and with longer hospital length of stay (LOS), besides having a variable cost per unit transfused [12,13].

Several studies have documented the variables associated with the need for blood transfusion in spine surgery, which include both patient-specific, i.e., age, gender, comorbidities, and surgery-related factors, i.e., operative time, multilevel procedures, deformity corrections [3,14]. Identifying factors associated with perioperative blood transfusion would allow for the early recognition of patients at greatest risk, and might improve current transfusion practice, leading to a more appropriate blood products allocation. However, no comprehensive studies characterizing the need for blood transfusion specifically on elective primary posterior lumbar fusion in centers of excellence for spine surgery are available. Thus, the purpose of this study was to retrospectively evaluate the perioperative blood transfusion need in patients undergoing elective primary lumbar posterior spine fusion and to determine predictors for red blood cell (RBC) transfusion in a high-volume center for spine surgery with approximately more than 650 procedures per year, half of which are at the lumbar level.

## 2. Material and Methods

### 2.1. Study Design, Setting, and Selection of Participants

The study was a retrospective cohort study in which the RBC transfusion ratio was evaluated in patients undergoing elective surgery for primary posterior lumbar spine fusion in the Neurosurgery I operative unit (O.U.), Neuro Center, at Humanitas Research hospital, Rozzano, Milan, Italy, over a period of 3 consecutive years between 1 January 2014 and 31 December 2016. The study was approved by the Institutional Review Board. At the time of hospital admission, each patient provided informed consent for the use of the data for research purposes. All patients undergoing elective surgery for lumbar spine fusion were included in the analyses. Patients admitted for urgent surgery (not elective) and/or with a previous lumbar fusion and/or with metastatic spine disease as cause for the surgery were excluded from the analyses. This was because these conditions are known to be associated with a higher risk of intraoperative bleeding [5].

### 2.2. Data Collection, Processing, and Outcomes

At the Humanitas Research hospital, all clinical data are prospectively collected and stored electronically in the data management system (Hospital, Lutech, Italy) in order to be retrieved at any time for clinical and research purposes. A retrospective database analysis was performed to identify all patients who underwent posterior lumbar spine fusion. Patients were identified using the Institutional procedural terminology code “8108” corresponding to surgery of posterior lumbar spine fusion. Screening of the electronic medical records to identify patients meeting inclusion criteria was performed by a medical doctor (GR) and an administrative responsible (DT). Patients’ demographics (age, gender, body mass index (BMI)), comorbidities (i.e., arterial hypertension, diabetes, ischemic cardiomyopathy, chronic use of anticoagulant/antiplatelet drugs) and American Society of Anesthesiologists (ASA) class, laboratory results (i.e., hemoglobin (HB), hematocrit (HCT) and coagulation parameters), occurrence and quantity (number of units) of RBC transfusion, operative time, and LOS, were retrieved for analysis. The coagulation parameters included platelets (PLT), partial prothrombin time (PTT), and international normalized ratio (INR). HB data were collected before the surgery, after completion of the surgery (day 0), and at 1, 2 and 3 days later. The main outcome was the occurrence of perioperative RBC transfusion, defined as any patient requiring at least 1 unit of RBC from the time of surgery up to hospital discharge.

### 2.3. Statistical Analysis

Categorical variables are presented as numbers and proportions, while continuous variables as median with interquartile range (IQR). Baseline characteristics by outcome occurrence (RBC transfusion) were compared by Chi-Square in case of categorical variables or by the non-parametric Mann–Whitney test for continuous non-normally distributed data. HB level changes over time were analyzed using the Wilcoxon signed rank test for paired data. Multivariable logistic regression was used to identify baseline factors that were predictors of RBC transfusion. All variables associated with the transfusion in the univariate analysis (*p* < 0.05) were included in the multivariable model. Odds ratios (OR) with the corresponding 95% CI were calculated and *p* values were considered statistically significant if they were less than 0.05. Prognostic accuracy of variables associated with the need for transfusion was evaluated by receiver operating characteristic curve (ROC) analyses and compared with Delong test. All statistical analyses were performed MedCalc Statistical Software version 17.7.2 (MedCalc Software bvba, Ostend, Belgium).

## 3. Results

**Figure 1:**
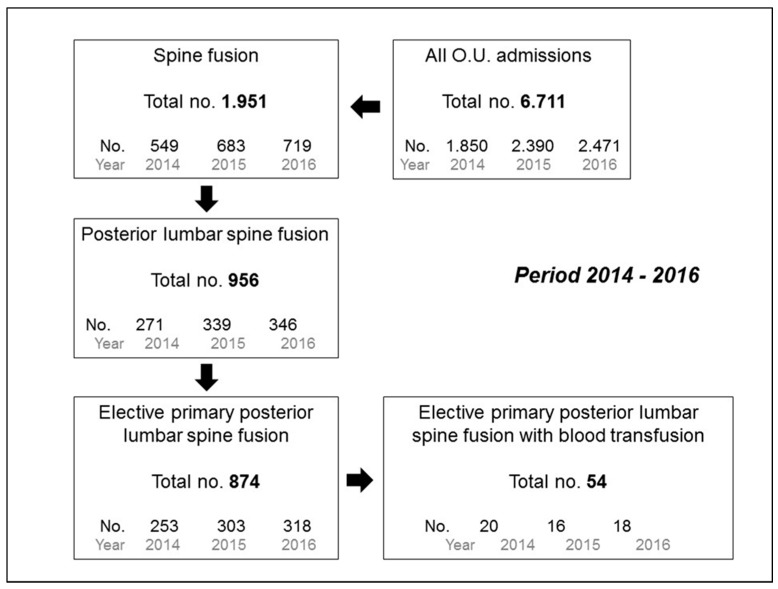
Flowchart showing the number of admissions at the operative unit (O.U.) Neurosurgery I, spine fusions, and posterior lumbar spine fusion surgeries over the three years of observation.

During the three years of observation, among the 6711 patients admitted to the O.U. Neurosurgey I, 5560 underwent a surgical procedure, 1951 of which were for spine fusion. Nine hundred-forty-eight of the above spine surgeries were specifically for posterior lumbar fusion and 874 of them were elective primary fusions (Figure 1). None of the patients underwent preoperative autologous blood donation nor intraoperative blood collection (Cell Saver). The majority of patients were female with a median age of 66 years and an ASA class of 2. Half of the patients presented a history of arterial hypertension, while lesser than 10% had diabetes and/or a previous ischemic cardiomyopathy. Sixteen percent of patients were assuming an anticoagulant/platelet therapy chronically, which was interrupted during the week prior to the surgery (Table 1). The median operative time was approximately 2 h. Pre-surgery HB was 14 g/dL; it then decreased of 2.6 g/dL during the first day after the surgery and of almost 5 g/dL after 3 days (Table 1).

Among the 874 patients undergoing elective primary posterior lumbar spine fusion, only 54 (6%) required RBC transfusion, which occurred on average on day 2 (1–3) after surgery. No difference in the surgical procedures, i.e., number of spinal segments instrumented and technique (all the procedures were “open”) were observed between the transfused and the non-transfused patients. Eighty-three percent of the transfused patients were female compared to the 60% of the non-transfused ones (*p* = 0.0008). Moreover, the transfused patients were older and had a higher ASA grade (*p* = 0.0002), with a significantly more frequent history of diabetes and use of anticoagulant/antiplatelet drugs, compared to the non-transfused ones (Table 2). Patients undergoing RBC transfusion presented a pre-surgery HB and HCT values significantly lower compared to those who did not receive the transfusion (*p* < 0.0001, Table 2). After surgery, the HB levels decreased in all patients (Table 1); however, in the transfused patients, the HB levels were consistently significantly lower compared to the non-transfused ones, already after completion of the surgery (9.9 g/dL vs. 12.1 g/dL, *p* < 0.0001) and during the subsequent 3 days (*p* < 0.0001, Table 2). At the moment of transfusion, median HB was 7.8 g/dL, and patients received a median of 2 RBC units.

The univariate odds ratios for prediction of RBC transfusion are reported in Table 3. Female gender, older age, higher ASA grade, presence of diabetes, use of anticoagulant/platelet drugs prior to the surgery, longer operative time, and lower pre-surgery HB level, were associated with the need for RBC transfusion. However, in the multivariate logistic regression, only a lower pre-surgery HB level (OR (95% CI) 2.84 (2.11–3.82), per 1 g/dL decrease), a higher ASA class (1.77 (1.03–3.05), per 1 grade increase) and a longer operative time (1.02 (1.01–1.02), per 1 min increase) were independently associated with the need for transfusion (Table 3).

**Figure 2:**
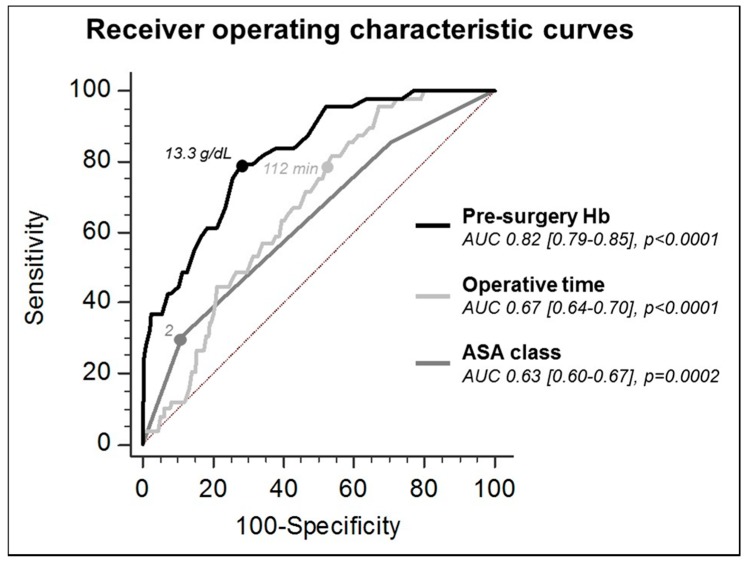
Receiver operator curves (ROC) and area under the curves (AUC) for pre-surgery hemoglobin (HB), American Society of Anesthesiologists (ASA) classification, and operative time for prediction of perioperative red blood cells transfusion.

The AUC of the ROC curves for prediction of RBC transfusion were: 0.63 for ASA grade (*p* = 0.0002); 0.67 for operative time (*p* < 0.0001); and 0.82 (*p* < 0.0001) for pre-surgery HB (*p* = 0.0001 vs. ASA class and *p* = 0.0004 vs. operative time) (Figure 2). The balanced cut off levels (sensitivity (Sn)/specificity (Sp)) for prediction of RBC transfusion were: 2 (Sn. 0.3/Sp. 0.9) for ASA class; 112 min (Sn. 0.8/Sp. 0.5) for operative time; and 13.3 g/dL (Sn. 0.8/Sp.0.7) for pre-surgery HB.

The LOS was in median 4 days in the whole population (Table 1). However, LOS was significantly longer in patients who received RBC transfusion compared to those not transfused (*p* < 0.0001, Table 2). RBC transfusion had an OR (95% CI) in predicting longer LOS of 8.04 (3.594–18.013) (*p* < 0.0001). In a multivariate model, adjusted for age, gender, BMI, ASA class, and operative time, being transfused was independently associated with longer LOS (OR (95% CI) 7.536 (2.925–19.410), *p* < 0.0001).

## 4. Discussion

This retrospective study demonstrated that in the instance of elective surgery for primary posterior lumbar fusion in a high-volume center for spine surgery, incidence of RBC transfusion was low, occurring in 1 patient every 16.5. Indeed, only 54 patients were transfused, over 874 undergoing elective surgery in a three-year period. Higher ASA class, longer operative time, and lower pre-surgery HB level were independent predictors of the need for perioperative transfusion. Finally, RBC transfusion was significantly associated with longer LOS.

Patients undergoing major spine surgery can experience significant intraoperative blood loss, with hemorrhage rates reported to be as high as 50 to 80% in adult patients [15,16]. Several factors may contribute to a direct blood loss, i.e., exposure of cancellous bone, stripping of skeletal muscles, extensive spinal instrumentation, transpedicular vertebrectomy, staged anterior-posterior approaches, and to an indirect blood loss, i.e., due to hypervascularization, altered blood coagulation profile, operative time, and intraoperative arterial hypertension [6,7,14,17,18].

The incidence of blood transfusion after spine surgery is quite variable and can be as high as 30% [12]. Considering specifically the lumbar fusion, in the Nationwide Inpatient Sample database, including data from approximately 1000 hospitals in the United States, an incidence of blood transfusion of 11% was reported in more than 1 million patient data analyzed over a period of six years) [19]. In the American College of Surgeons National Surgical Quality Improvement Program (ACS-NSQIP), another United States database which captures data from over 400 participating hospitals, among the 4223 primary posterior lumbar fusion procedures over a period of three years, 16.7% required blood transfusion [4]. Similarly, in the nationwide Canadian network, 18% of the 772 patients undergoing posterior lumbar fusion over an eight-year period had blood transfusion [20]. In our O.U. we observed a lower incidence of RBC transfusion, which occurred in only 6% of 874 patients subjected to elective surgery. Such a low incidence may be explained as a consequence of the much higher volume of spinal procedures performed in our O.U., having achieved more than 700 cases per year, in comparison to a rate ranging from 4 to 167 cases per year in the above National databases, which included a variety of low, middle, and high hospital caseloads [4,19,20]. Likely, the high rate of spine surgeries per year in our O.U. accounted for a continuous procedure optimization with concurrent complications reduction. In addition, in our analyses, only elective primary lumbar fusion mainly due to degenerative spine disease, i.e., spondylolisthesis, were included, excluding urgent procedures due to trauma, cases due to metastatic spinal tumor, and revisions of a previous lumbar fusion—conditions known to be associated with at least a three-fold higher risk of massive bleeding, due to either a tumor-related hypervascularization and/or more extensive spinal instrumentations [5,21,22].

In accordance with earlier reports, in our population, factors associated with perioperative blood transfusion were older age, female gender, higher ASA grade, presence of diabetes, use of anticoagulant/platelet drugs, longer operative time, and low pre-surgery HB levels. However, in the multivariate model, only ASA grade, operative time, and pre-surgery HB were confirmed as independent predictors of RBC transfusion [4,12,19,20]. Thus, it has been reported that older patients were more likely to receive a blood transfusion than middle-aged patients and that as the number of comorbidities increased, the probability of blood transfusion increased as well [12,19]. When patients had more than 85 years and four or more comorbidities, a triple risk to receive blood transfusion has been described [19]. Similarly, an ASA score ≥2 or ≥3 had up to 6 or 18-fold greater probability to receive blood transfusion respectively, compared to a score <2 or <3 [12,19,20,23]. Indeed, in our population, an age >66 years accounted for a 2.1-fold higher risk of blood transfusion, and the need for RBC transfusion increased of 77% for each unit increase in the ASA grade, accounting overall for a 2.3-fold higher incidence of transfusion for an ASA >2. These relationships have been explained by the concurrent presence of comorbidities in aged patients, for which physicians prefer to maintain higher HB levels in order to improve oxygen delivery to the tissues [24,25]. However, this was not the case in our population, since the median HB was already 7.8 g/dL at the time of transfusion. The greater use of medications interfering with coagulation or platelet function in aged people with comorbidities has been hypothesized as an additional explanation [24,25]. Indeed, our data confirmed past research that suggested such treatments significantly increased the risk of bleeding, either intraoperatively and/or from postoperative blood drainages, in patients undergoing lumbar fusion, despite discontinuation of therapy at least 1 week preoperatively [26,27,28].

Extended surgical time has been also previously associated with transfusion requirements following primary lumbar fusion, with an increase in the odds of transfusion of 4.2% for each 60-min increase, probably related to a greater complexity of the procedures, with extensive bone exposure and instrumentation [4,5,6,7,12,14,17,18,19,20]. This relationship was confirmed in our large population, in which the need for blood transfusion increased by almost 1.6% for each minute of surgery, leading to almost a three-fold rise for procedures lasting more than 2 h.

Among all the factors associated with the need for perioperative blood transfusion in our population, pre-surgery HB level, however, has appeared as the most predictive one. Indeed, a 2.8-fold increase in the risk to receive an RBC transfusion was observed for each 1 g/dL decrease in HB, and a pre-surgery HB <13.3 g/dL accounted for an odds for transfusion of 6.7. This predictive value of 13.3 g/dL is quite similar to that of 13.6 earlier reported in a smaller population [23]. Likely, pre-surgery HB levels can be also involved in the higher requirement of RBC transfusion in females, who are known to be more inclined to anemia [19]. In our population, in fact, the female gender was associated with a higher incidence of blood transfusion only in the univariate model, but the association was lost in the multivariate one.

Indeed, preoperative anemia, together with the amount of perioperative HB drop, were shown to be independent risk factors for increased morbidity, mortality, and longer LOS, with overall increased healthcare costs [4,6,12,29,30,31,32,33]. Thus, the goal for an optimal pre-surgery patient’s preparation would be to detect a condition of anemia or borderline HB levels as early as possible before the scheduled surgery in order to provide correction with treatments directed to increase the RBC mass, i.e., administration of iron, vitamin B12, folic acid, and erythropoietin [34,35,36]. Optimizing the RBC mass before the spine surgery may be a pillar for an optimized patient blood management. However, even if preoperative anemia is adequately corrected prior to surgery, there is still the risk of massive bleeding during spine surgery. In these instances, antifibrinolytic drugs have been used efficiently to decrease perioperative blood loss, up to 49%, and transfusion requirements, up to 80% in some instances [37,38,39].

Thus, at the time of patient’s preparation to the surgery, paying attention to factors associated with the need for perioperative blood transfusion, like the ones emerged from our study, i.e., ASA class, expected operative time, and baseline HB level, may provide guidance to determine which patients will benefit from preoperative optimization of the RBC mass, and in the planning for perioperative invasive monitoring, intravenous access, blood product availability, and eventually administration of anti-fibrinolytics [14,40]. Efforts to minimize perioperative administration of blood products might decrease the costs associated with blood transfusion that range between 700–1200 US dollars for administration of one RBC unit, plus additional costs up to 1000 US dollars for treatment of associated side effects [13,41,42]. Indeed, when high-risk patients were identified and an optimization protocol was initiated, a significant reduction in perioperative transfusions was reported, with a cost of approximately 850 US dollars, which represented a clearly favorable approach as it was still cheaper than one unit of RBC and its side effects [36,40]. Another interventional cohort study demonstrated that within 1 year from the introduction of a program of patient blood management, transfusions of all allogeneic blood products per 1000 patients was reduced by 27% with a cost reduction of over 2 million US dollars on a hospital level [42]. In our O.U., the overall cost for RBC transfusion in the 54 patients over the three years of observation amounted to approximately 28,000 euros without considering other costs related to the longer LOS and treatment of complications.

We need to acknowledge several limitations in the interpretation of our findings. First, this was a single-center retrospective study and thus a selection bias is present. Nevertheless, all patients undergoing elective primary posterior lumbar spine fusion were included in the analyses. Moreover, the large number of patients included and the high-quality data have advantages that outweigh this limitation. Indeed, our O.U. presented a high-volume of spine fusion surgeries, such that our population may be considered sufficiently representative to make results translatable to other centers. Second, we did not report the amount of perioperative bleeding; however, we have included data on HB from day 0, at the end of the surgery, and then daily up to day 3 after the surgery. Finally, we did not evaluate additional preventive measures to decrease the intraoperative bleeding, i.e., anti-fibrinolytic therapy, although these might have occasionally occurred during high-risk spinal procedures.

## 5. Conclusions

This retrospective study demonstrated that in the instance of elective surgery for primary posterior lumbar fusion in a high-volume center for spine surgery, incidence of red blood cell transfusion was low, occurring in 1 patient every 16.5. Higher American Society of Anesthesiologists class, longer operative time, and lower pre-surgery hemoglobin level, were independent predictors for the need of perioperative red blood cell transfusion. Finally, a low pre-surgery hemoglobin level was the most significant predictor of perioperative red blood cell transfusion.

## Figures and Tables

**Table 1:** Whole population description.

Population, n	874
Age, year	66 (56–73)
Male, n (%)	333 (38)
BMI, kg/m^2^	26 (23–29)
Comorbities, n (%)	
*Ischemic cardiomyopathy*	42 (5)
*Arterial hypertension*	421 (49)
*Diabetes*	82 (10)
*Use of anticoagulant/antiplatelet drugs*	138 (16)
ASA, class	2 (1–2)
HB pre-surgery, g/dL	14 (13.1–14.9)
HCT pre-surgery, %	42 (40–45)
INR pre-surgery, ratio	1 (0.96–1.04)
PTT pre-surgery, ratio	1.01 (0.95–1.07)
Platelets, n*10^3^/mm^3^	232 (198–270)
Operative time, min	117 (96–140)
HB on day 0 after surgery, g/dL	11.6 (10.7–12.7) *
HB on day 1 after surgery, g/dL	11.4 (10.4–12.4) *§
HB on day 2 after surgery, g/dL	10.3 (9.4–11.2) *§
HB on day 3 after surgery, g/dL	9.2 (8.6–10.1) *§
Transfused patients, n (%)	54 (6)
LOS, day	4 (4–5)

Data are reported as median (interquartile) BMI, body mass index; ASA, American Society of Anesthesiologists classification; HB, hemoglobin; HCT, hematocrit; INR, International normalized ratio (prothrombin time); PTT, partial thromboplastin time; LOS, length of stay.* *p* < 0.01 vs. HB pre-surgery; § *p* < 0.01 vs. HB in the preceding day.

**Table 2:** Comparison between transfused and not transfused patients.

	Transfused (*n* = 54)	Not Transfused (*n* = 820)	*p* Value
Male, n (%)	9 (17)	324 (40)	0.00
Age, year	71 (63–74)	66 (55–73)	<0.01
BMI, kg/m^2^	25 (23–28)	26 (23–29)	0.22
Comorbities, n (%)			
*Ischemic cardiomyopathy*	4 (8)	38 (5)	0.34
*Arterial hypertension*	29 (56)	392 (49)	0.34
*Diabetes*	11 (20)	71 (9)	0.01
*Use of anticoagulant/antiplatelet drugs*	14 (26)	124 (16)	0.04
ASA, class	2 (2–3)	2 (1–2)	0.00
HB pre-surgery, g/dL	12.7 (11.3–13.3)	14 (13.2–14.9)	<0.01
HCT pre-surgery, %	38 (35–40)	43 (40–45)	<0.01
INR pre-surgery, ratio	1 (0.95–1.04)	1 (0.96–1.04)	0.98
PTT pre-surgery, ratio	0.99 (0.94–1.06)	1.01 (0.95–1.07)	0.43
Platelets, n*10^3^/mm^3^	235 (203–266)	232 (198–270)	0.88
Operative time, min	133 (114–151)	117 (95–139)	0.00
HB on day 0 after surgery, g/dL	9.9 (9.2–10.8) *	12.1 (11.2–13.1) *	<0.01
Hb on day 1 after surgery, g/dL	9.1 (8.8–9.9) *§	11.5 (10.6–12.4) *§	<0.01
HB on day 2 after surgery, g/dL	8.3 (7.8–9.1) *§	10.4 (9.6–11.4) *§	<0.01
HB on day 3 after surgery, g/dL	8.5 (7.9–9.6) *#	9.4 (8.7–10.3) *§	<0.01
HB at transfusion, g/dL	7.8 (7.4–7.9) *	N.A.	N.A.
RBC units transfused, n	2 (2–2)	N.A.	N.A.
LOS, day	6 (5–7)	4 (4–5)	<0.01

Data are reported as median (interquartile); comparisons performed with 2-tail Mann-Whitney test. BMI, body mass index; ASA, American Society of Anesthesiologists classification; HB, hemoglobin; HTC, hematocrit; INR, International normalized ratio (prothrombin time); PTT partial thromboplastin time; RBC, packed red blood cell; LOS, length of stay. * *p* < 0.01 vs. HB pre-surgery; # *p* < 0.05 and § *p* < 0.01 vs. HB in the preceding day.

**Table 3:** Univariate and multivariable logistic regression models for prediction of red blood cells transfusion.

	Univariate			Multivariable		
	OR	95% CI	*p* Value	OR	95% CI	*p* Value
Gender, female	3.266	1.575–6.772	0.01	1.342	0.554–3.249	0.52
Age	1.037	1.009–1.066	0.01	1.022	0.986–1.059	0.24
HB pre-surgery	2.937	2.231–3.865	<0.01	2.838	2.108–3.820	<0.01
ASA class	2.431	1.527–3.870	0.00	1.773	1.032–3.046	0.04
Presence of diabetes	2.627	1.297–5.319	0.01	1.625	0.660–3.999	0.29
Use of anticoag/platelets	1.908	1.032–3.54	0.05	1.713	0.782–3.754	0.18
Operative time	1.014	1.007–1.021	0.00	1.016	1.007–1.024	0.00

OR, odds ratio; ASA, American Society of Anesthesiologists classification; HB, hemoglobin.
